# Block of the Mevalonate Pathway Triggers Oxidative and Inflammatory Molecular Mechanisms Modulated by Exogenous Isoprenoid Compounds

**DOI:** 10.3390/ijms15046843

**Published:** 2014-04-22

**Authors:** Paola Maura Tricarico, Giulio Kleiner, Erica Valencic, Giuseppina Campisciano, Martina Girardelli, Sergio Crovella, Alessandra Knowles, Annalisa Marcuzzi

**Affiliations:** 1 Department of Medicine and Surgery and Health, University of Trieste, Piazzale Europa, 1, 34128 Trieste, Italy; E-Mails: tricaricopa@gmail.com (P.M.T.); g.campisciano@gmail.com (G.C.); sergio.crovella@burlo.trieste.it (S.C.); 2 Institute for Maternal and Child Health IRCCS “Burlo Garofolo”, 34137 Trieste, Italy; E-Mails: giulio.kleiner@burlo.trieste.it (G.K.); erica.valencic@gmail.com (E.V.); martina.girardelli@burlo.trieste.it (M.G.); alessandra.knowles@burlo.trieste.it (A.K.)

**Keywords:** isoprenoids, mevalonate, inflammation, phytol, lycopene

## Abstract

Deregulation of the mevalonate pathway is known to be involved in a number of diseases that exhibit a systemic inflammatory phenotype and often neurological involvements, as seen in patients suffering from a rare disease called mevalonate kinase deficiency (MKD). One of the molecular mechanisms underlying this pathology could depend on the shortage of isoprenoid compounds and the subsequent mitochondrial damage, leading to oxidative stress and pro-inflammatory cytokines’ release. Moreover, it has been demonstrated that cellular death results from the balance between apoptosis and pyroptosis, both driven by mitochondrial damage and the molecular platform inflammasome. In order to rescue the deregulated pathway and decrease inflammatory markers, exogenous isoprenoid compounds were administered to a biochemical model of MKD obtained treating a murine monocytic cell line with a compound able to block the mevalonate pathway, plus an inflammatory stimulus. Our results show that isoprenoids acted in different ways, mainly increasing the expression of the evaluated markers [apoptosis, mitochondrial dysfunction, nucleotide-binding oligomerization-domain protein-like receptors 3 (NALP3), cytokines and nitric oxide (NO)]. Our findings confirm the hypothesis that inflammation is triggered, at least partially, by the shortage of isoprenoids. Moreover, although further studies are necessary, the achieved results suggest a possible role for exogenous isoprenoids in the treatment of MKD.

## 1. Introduction

**Figure 1.**
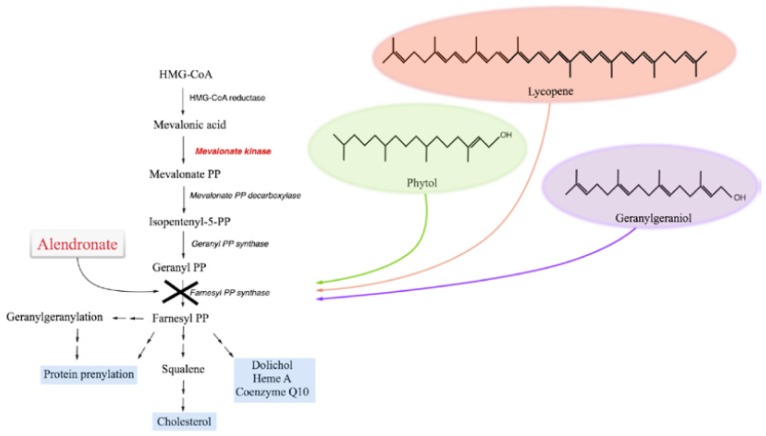
Schematic representation of the mevalonate pathway. Compounds used in the experiments are indicated alongside the pathway: alendronate was used to biochemically block the pathway, while isoprenoids were used to rescue it.

The mevalonate pathway is a crucial metabolic process finalized to produce a single major sterol species, cholesterol. Given its manifold physiological functions, cholesterol metabolism is of fundamental importance throughout the human body. It is therefore easily understood that the deregulation of this molecule lies at the root of many diseases, such as the Smith-Lemli-Opitz syndrome, caused by a defect in the conversion of post-squalene compounds in cholesterol, or other peroxisomal disorders, characterized by low plasma cholesterol levels [1,2]. Another example which highlights the importance of this metabolic pathway, is represented by an auto-inflammatory orphan disease, called mevalonate kinase deficiency (MKD, OMIM #610377). MKD is caused by mutations affecting the gene (*MVK*, 12q24) encoding the second enzyme of the mevalonate pathway (mevalonate kinase, MK), and is characterized by the consequent shortage of intermediate compounds, as well as final products, of the metabolic route (Figure 1) [3].

Patients suffering from MKD exhibit recurrent episodes of fever and associated inflammatory symptoms. Moreover, in the most severe forms, patients display developmental delay, dysmorphic features, ataxia, cerebellar atrophy, psycho-motor retardation, and may die in early childhood [4]. Furthermore, while the symptoms subside between fever attacks, increased levels of acute-phase reactants and pro-inflammatory cytokines are detected in the serum of patients during the acute episodes [5]. In detail, as demonstrated by *ex vivo* studies, peripheral blood mononuclear cells from MKD patients produce large amounts of pro-inflammatory cytokines, such as IL-1β, IL-6, TNF-α, in response to lipopolysaccharide (LPS) [6]. It is commonly assumed that caspase-1-dependent IL-1β, derived from the activation of the NALP3-inflammasome (NALP: nucleotide-binding oligomerization-domain protein-like receptors), represents the major cytokine responsible for the systemic inflammatory events observed in MKD patients [7,8]. In these patients, although the metabolic pathway is deregulated (and in contrast to all expectations), plasma levels of cholesterol are on the whole normal [4].

This clinical aspect may be explained by physiological homoeostasis, which is able to create compensatory mechanisms so that the resulting cholesterol levels are barely altered, even if this is detrimental for other intermediate compounds [3].

The most promising hypothesis to explain inflammation is based on the evidence that blocking the biochemical pathway induces a lack of isoprenoid compounds [9]. Indeed, our research group recently demonstrated that natural isoprenoids (geraniol, farnesol, geranylgeraniol, menthol and others) are able to rescue the MKD-inflammatory phenotype, by affecting the production of cytokines (IL-1β, IL-18, TNF-α) and inflammatory markers [serum amyloid A (SAA), and apoptosis] both *in vitro* and *in vivo* [10].

In addition, Tricarico *et al.* showed that, in MKD, programmed cell death is supported by a balance between apoptosis and pyroptosis, with the involvement of mitochondrial damage [11]. These results strengthen the hypothesis introduced by Celec *et al.*, according to which the lack of natural isoprenoids would be linked to the most severe forms of the disease [12].

Recently, an exhaustive study [13] demonstrated that the production of IL-1β originates from defects in autophagy. In particular, the hyper-secretion of pro-inflammatory cytokines is triggered by reactive oxygen species. IL-1β production is associated with an oxidized redox status of monocytes, and subsequently involved the decrease of mitochondrial stability and, finally, in autophagosomal degradation. Moreover, this mechanism seems to be a starter system for the activation of the NALP3 inflammasome, which in turn contributes to the augmented production of IL-1β.

Hence, the objective of our study is firstly to assess the anti-inflammatory activity of isoprenoids such as geranylgeraniol, phytol, and lycopene, administered in a MKD cellular model, and, secondly, to establish if this anti-inflammatory activity is related to their already known anti-oxidant properties [14–16].

The ability of these compounds to rescue the MKD pathway and revert the inflammatory phenotype was assessed through the evaluation of cellular damage (programmed cell death) mitochondrial dysfunction (variations in the mitochondrial membrane potential), and inflammatory and oxidative markers (NALP3, cytokines and nitric oxide). The assessments of the mechanisms by which these natural compounds achieve their anti-inflammatory effects on the biochemical block of the metabolic pathway is crucial for understanding the pathogenesis of MKD and subsequent discoveries of new targets for potential drugs to be designed for this orphan disease.

## 2. Results and Discussion

### 2.1. The Block of the Mevalonate Pathway Induces Programmed Cell Death (PCD) and Mitochondrial Dysfunction

Previous studies have suggested that the deregulation of the mevalonate pathway induces activation of inflammatory mechanisms, such as cytokine production, which is in accordance with what has been seen in MKD patients [17]. In order to reproduce a pathological condition similar to MKD, we decided to induce a biochemical block of the pathway and introduce an inflammatory stimulus, using alendronate and LPS, respectively.

This is in agreement with previous data from the literature, which report that a double treatment [Ald (Alendronate) + LPS] is necessary to induce a significant increase of both parameters, compared to the single compounds [17]. The block obtained using Ald, followed by the inflammatory stimulus, dramatically increased both apoptosis (expressed as % of A+) (Ald + LPS: 69.2 ± 7.9; Ald: 41.7 ± 9.5; LPS: 10.7 ± 5.3; Untreated: 6.3 ± 2.4) and mitochondrial dysfunction [expressed as mean fluorescence intensity (MFI)] (Ald + LPS: 116.8 ± 7.8; Ald: 154.7 ± 12.9; LPS: 448.0 ± 80.6; Untreated: 457.7 ± 50.7).

**Figure 2.**
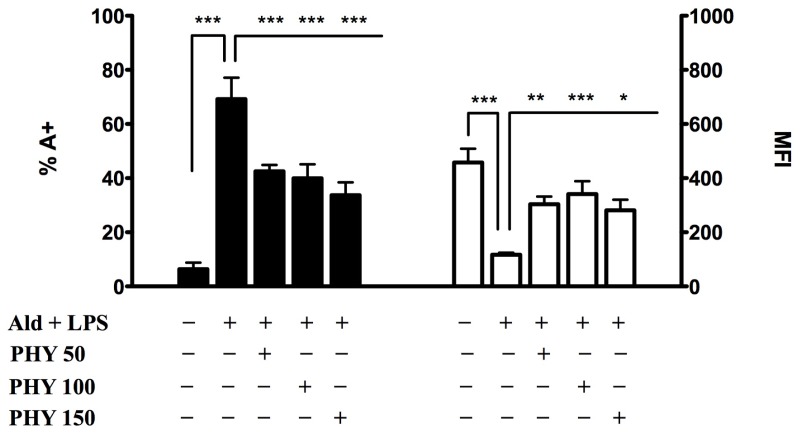
Phytol (PHY) reduces programmed cell death (PCD) and mean fluorescence intensity (MFI). Raw 264.7 cells were incubated with 100 μM Ald and at the same time with different concentrations of Phytol (50, 100 and 150 μM) for 20 h and then with 10 μg/mL LPS for an additional 24 h. On the left PCD: bars represent the mean % of Annexin V positive cells (A+) ± standard deviation (SD) of three independent experiments. On the right MFI: bars represent the mean fluorescent intensity of Rhodamine 123 ± SD of three independent experiments. Data analyses were performed with one-way ANOVA and Bonferroni correction comparing Ald (Alendronate) + LPS (lipopolysaccharide) treated cells with other experimental conditions; * *p* < 0.05; ** *p* < 0.01; *** *p* < 0.001.

**Figure 3.**
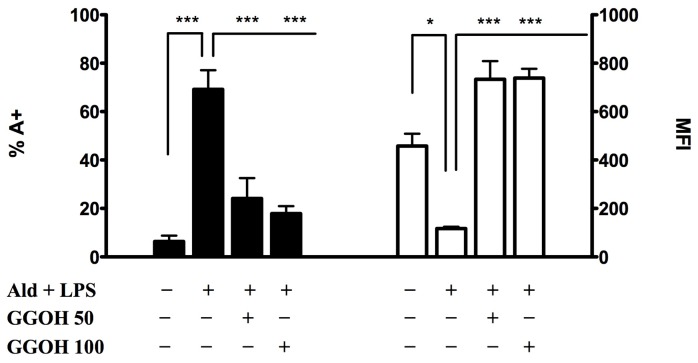
Geranylgeraniol (GGOH) significantly affected both PCD and MFI. Raw 264.7 cells were incubated with 100 μM Ald and, at the same time, with different concentrations of geranylgeraniol (50–100 μM) for 20 h and then with 10 μg/mL LPS for an additional 24 h. On the left PCD: bars represent the mean % of Annexin V positive cells (A+) ± standard deviation (SD) of three independent experiments. On the right MFI: bars represent the mean fluorescent intensity of Rhodamine 123 ± SD of 3 independent experiments. Data analyses were performed with one-way ANOVA and Bonferroni correction comparing Ald + LPS treated cells with other experimental conditions; * *p* < 0.05; *** *p* < 0.001.

**Figure 4.**
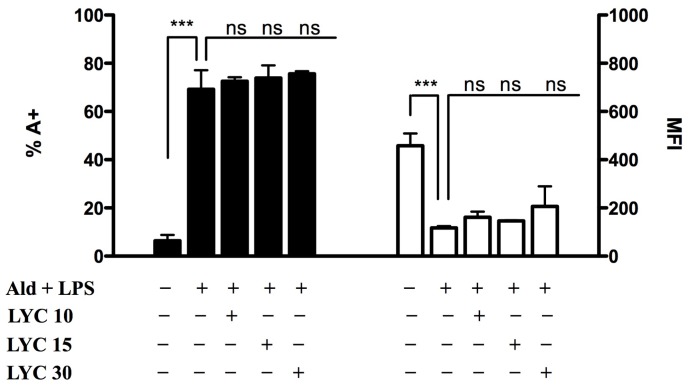
Lycopene (LYC) does not affect either PCD or MFI. Raw 264.7 cells were treated for 2 h at 37 °C with different concentrations of Lycopene (10, 15 and 30 μM) and then stimulated with 100 μM Ald for 20 h and with 10 μg/mL LPS for an additional 24 h. On the left PCD: bars represent the mean % of Annexin V positive cells (A+) ± SD of three independent experiments. On the right MFI: bars represent the mean fluorescent intensity of Rhodamine 123 ± standard deviation (SD) of 3 independent experiments. Data analyses were performed with one-way ANOVA and Bonferroni correction comparing Ald + LPS treated cells with other experimental conditions; *** *p* < 0.001; ns: non significant.

Under these experimental conditions, we then studied the effects of selected isoprenoid compounds, such as phytol (PHY 50–100–150 μM) (Figure 2), geranylgeraniol (GGOH 50–100 μM) (Figure 3) and lycopene (LYC 10–15–30 μM) (Figure 4). Different ranges of isoprenoids’ concentrations were tested, in accordance with previously reported studies [18–20].

#### 2.1.1. Phytol

The percentage of programmed cell death (PCD) was significantly reduced after PHY treatments, irrespective of the concentration used (PHY 50 + Ald + LPS: 42.6 ± 2.3, *p* < 0.001; PHY 100 + Ald + LPS: 39.9 ± 5.1, *p* < 0.001; PHY 150 + Ald + LPS: 33.7 ± 4.7, *p* < 0.001), compared to Ald + LPS. Comparisons within the same group (Ald + LPS) showed that MFI values were significantly increased after treatment with PHY 50 and 100 μM, and less markedly increased after PHY 150 μM treatment (PHY 50 + Ald + LPS: 304.0 ± 27.8, *p* < 0.01; PHY 100 + Ald + LPS: 341.7 ± 47.1, *p* < 0.001; PHY 150 + Ald + LPS: 281.7 ± 38.7, *p* < 0.05) (Figure 2).

#### 2.1.2. Geranylgeraniol

All tested concentrations of geranylgeraniol (GGOH) significantly affected the evaluated markers: percentages of PCD were dramatically decreased (GGOH 50 + Ald + LPS: 24.1 ± 8.5, *p* < 0.001; GGOH 100 + Ald + LPS: 17.9 ± 3.1, *p* < 0.001), while MFI values were greatly increased (GGOH 50 + Ald + LPS: 734.0 ± 74.9, *p* < 0.001; GGOH 100 + Ald + LPS: 738.5 ± 38.9, *p* < 0.001) (Figure 3).

#### 2.1.3. Lycopene

None of tested concentrations of LYC was able to affect either PCD or MFI, when compared to Ald + LPS (PCD: LYC 10 + Ald + LPS: 72.5 ± 1.6; LYC 15 + Ald + LPS: 73.8 ± 5.3; LYC 30 + Ald + LPS: 75.6 ± 1.1; MFI: LYC 10 + Ald + LPS: 161.5 ± 93.3; LYC15 + Ald + LPS: 146.5 ± 2.1; LYC30 + Ald + LPS: 206.5 ± 82.7) (Figure 4).

Following Ald + LPS treatment, we observed an increase in apoptosis and a decrease in MFI, the latter being inversely proportional to mitochondrial damage. These results confirm that the blockade of the mevalonate pathway determines mitochondrial damage correlated to the intrinsic apoptosis, as reported in previous studies [21]. Phytol and geranylgeraniol limited the mitochondrial dysfunction, thus protecting cells from apoptosis. This suggests that these two isoprenoids are able to enter the mevalonate pathway and restore the damage caused by the induced biochemical block in our cell model.

Surprisingly, lycopene did not increase either PCD or MFI values, as instead did phytol and geranylgeraniol. Honestly, we cannot at the moment explain the different effects of the tested isoprenoids on apoptosis and mitochondrial damage.

Since PCD and MFI values showed a significant decrease at all concentrations of PHY and GGOH, PHY 100 μM and GGOH 100 μM were chosen for all subsequent experiments, being the smallest doses with the maximum effect. Given that instead LYC didn’t shown any significant modulation of the inflammatory markers, an intermediate dose, LYC 15 μM, was chosen. MFI values were less markedly increased after PHY 150 μM treatment (PHY 50 + Ald + LPS: 304.0 ± 27.8, *p* < 0.01; PHY 100 + Ald + LPS: 341.7 ± 47.1, *p* < 0.001; PHY 150 + Ald + LPS: 281.7 ± 38.7, *p* < 0.05)

### 2.2. Effects of Isoprenoids on Cytokine Profile

**Figure 5.**
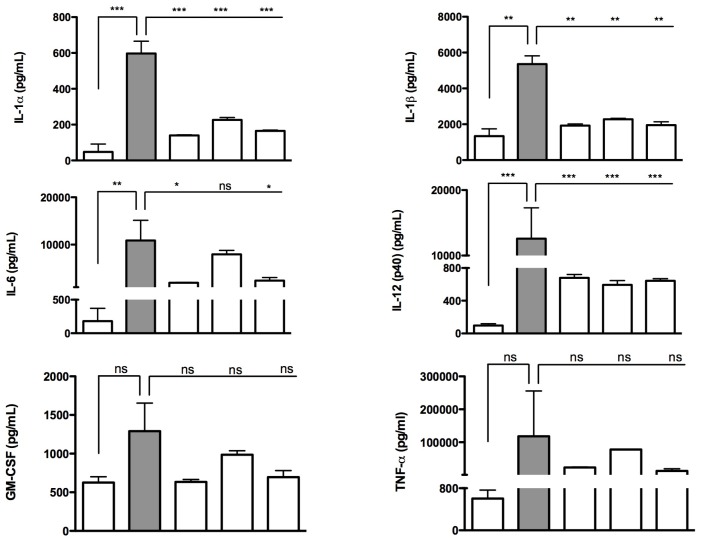

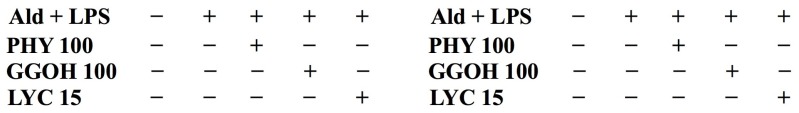
Deregulated cytokines after treatments on Raw 264.7 cells. Phytol 100 μM (Phy 100) and geranylgeraniol 100 μM (GGOH 100) were dispensed after the alendronate (Ald 100 μM) + lipopolysacchardide (LPS 10 μg/mL) treatment. Lycopene 15 μM (LYC 15) was given as a pre-treatment 2 h before the Ald + LPS treatment. The concentration values of cytokines (pg/mL) were obtained from experiments performed in triplicate. IL-1α, IL-1β, IL-6 and IL-12 were significantly modulated with isoprenoids treatments. * *p* < 0.05; ** *p* < 0.01; *** *p* < 0.001; ns = not significant after one way ANOVA test followed by the Bonferroni correction for multiple comparisons.

The pro-inflammatory cytokines IL-1α, IL-1β, IL-6 and IL-12 (p40) were significantly up-regulated after Ald + LPS treatment, compared to untreated conditions, whereas PHY 100 μM, GGOH 100 μM and LYC 15 μM treatments were able to significantly reduce the release of these cytokines. While not undergoing significant changes, also granulocyte-macrophage colony-stimulating factor (GM-CSF) and TNF-α displayed trends consistent with the other evaluated cytokines (Figure 5).

These findings are sustained by several studies in literature that report the involvement of IL-1α, IL-1β, IL-6 and IL-12 (p40) in the inflammatory processes of MKD [6,22,23]. Moreover, even if less marked, we also observed the involvement of IL-12 (p40) and GM-CSF, which also exert a pro-inflammatory activity and are able to coordinate the subsequent immune response [24,25].

In particular, the IL-1 family is strongly believed to play a fundamental role in MKD inflammatory processes; indeed, it represents the major cytokine responsible for the systemic inflammatory effects observed in patients suffering from this disease [23].

PHY, GGOH and LYC could prove to be very important anti-inflammatory molecules, inasmuch as they are able to modulate the main inflammatory markers of MKD, *i.e.*, IL-1α, IL-1β, IL-6, and TNF-α.

### 2.3. Inflammasome (NALP3) Expression in the Presence of Anti-Inflammatory/Anti-Oxidant Compounds

**Figure 6.**
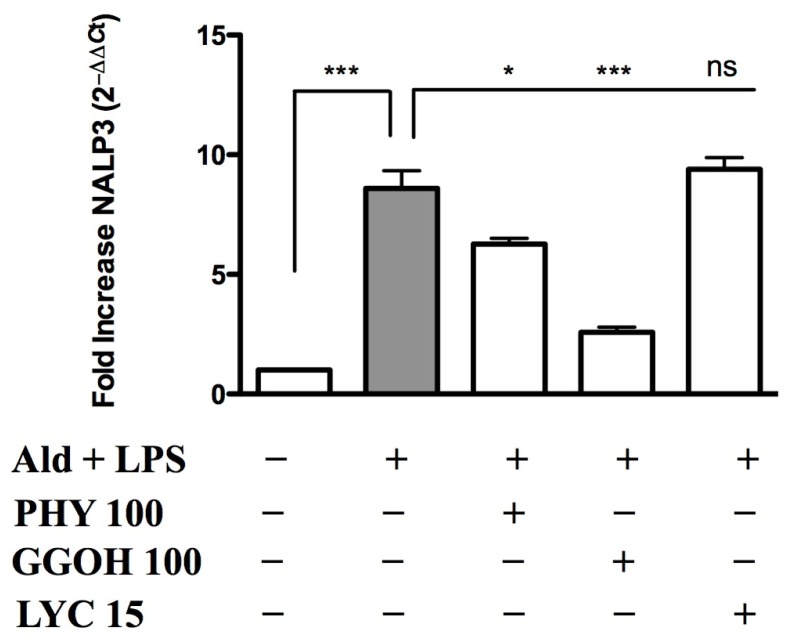
NALP3-inflammasome expression in Raw 264.7 cells. Expression of NLRP3/NALP3 was measured after Alendronate (100 μM) + LPS (10 μg/mL) treatment alone or in combination with phytol 100 μM, GGOH 100 μM or LYC 15 μM treatments. Analyses were performed using real time quantitative polymerase chain reaction (PCR), and results were normalized to *ACTB* expression. Expression of untreated cells was normalized to 1. Expression data for the three experiments are reported as 2^−ΔΔ^*^C^*^t^ average ± SD, in which ΔΔ*C*_t_ = Δ*C*_t__stimulated H*C*–Δ*C*_t__RH*C*. Data analyses were performed with one-way ANOVA and Bonferroni correction comparing Ald + LPS treated cells with the other experimental conditions; * *p* < 0.05; *** *p* < 0.001; ns: non significant.

Under Ald + LPS-treated conditions, NALP3-inflammasome expression levels significantly increased (8.6 ± 0.7, *p* < 0.001) compared to untreated conditions, which were given a reference value of 1. The treatment with PHY 100 μM (6.3 ± 0.2, *p* < 0.05) and GGOH 100 μM (2.6 ± 0.2, *p* < 0.001) significantly decreased NALP3 expression, compared to the treatment with Ald + LPS alone. On the other hand, LYC 15 μM was not able to significantly revert the effects of Ald + LPS treatment on the inflammasome expression (9.4 ± 0.5) (Figure 6).

NALP3-inflammasome activation is responsible for IL-1β production, which represents the major cytokine responsible for the systemic inflammatory events observed in MKD patients [7,8].

An increase in the expression of NALP3 was previously reported in studies conducted both in monocytes isolated from MKD patients and in *in vivo* models of MKD [26,27].

The biochemical blockade of the mevalonate pathway leads to an increase of NALP3 expression; this up-regulation is consistent with the increasing trend of IL-1β secretion. The administration of geranylgeraniol or phytol led to a significant decrease in NALP3 expression, which is in turn linked to a diminished secretion of IL-1β; it is thus possible to assume that phytol and geranylgeraniol exert an anti-inflammatory activity, and are able to rescue the inflammatory phenotype in this MKD cellular model.

### 2.4. Nitric Oxide Release after Blocking the Pathway and Rescuing it with Exogenous Isoprenoids

**Figure 7.**
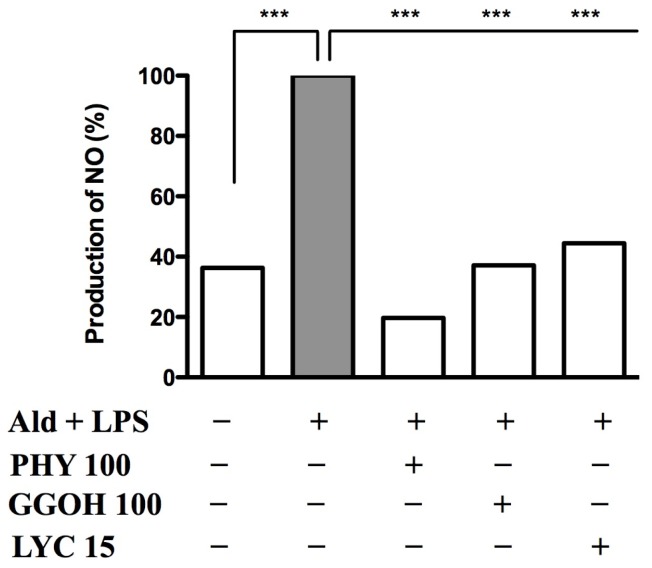
PHY (100 μM), GGOH (100 μM) and LYC (15 μM) reduced NO production in Raw 264.7 cell line stimulated with Alendronate (100 μM) + LPS (10 μg/mL). Data are expressed as percentage of NO production with respect to Ald + LPS treated cells (100%) (*n* = 3). Data analyses were performed with one-way ANOVA and Bonferroni correction comparing Ald + LPS treated cells with other experimental conditions; *** *p* < 0.001.

Nitric oxide (NO) values were evaluated in Raw 264.7 cells treated with Ald + LPS. This treatment induced a significant production of NO when compared to untreated conditions (Ald + LPS: 100%; untreated: 36.3%), in accordance with previous studies [17]. We then evaluated the effects of PHY, GGOH and LYC using this cellular model, in which the inhibition of the mevalonate pathway was able to amplify LPS-induced inflammation. All tested compounds reduced NO production (PHY: 19.7%; GGOH: 37.1%; LYC: 44.4%) compared to Ald + LPS (100%). As shown in Figure 7, all three isoprenoids were able to significantly reduce the Ald + LPS-induced NO production.

As a result of the biochemical block of the mevalonate pathway and the subsequent pro-inflammatory stimulus, we detected an increase of the oxidative marker NO. The exogenous isoprenoid compounds, which intervene along the mevalonate pathway, were able to determine a significant decrease of this oxidative marker. Since NO is an important oxidative marker in inflammatory processes, and its over-production indicates oxidative stress [28,29], these results confirm the anti-oxidant activity of phytol, geranylgeraniol and lycopene.

## 3. Experimental Section

Where not specified, reagents were purchased from Sigma-Aldrich (Milan, Italy).

### 3.1. Chemicals

Lipopolysaccharide (LPS, *E. coli*-serotype 055:B5, 1 mg/mL stock in H_2_O). Alendronate (Ald) was dissolved in saline solution (Diaco SpA, Trieste, Italy). Isoprenoid compounds: geranylgeraniol (GGOH) solubilized in ethanol 100% to form a stock solution of 0.6 M GGOH, phytol (PHY), and lycopene (LYC) solubilized in tetrahydrofuran stabilized with 0.025% BHT to form a stock solution of 10 mM LYC. All isoprenoids were then diluted in culture media at desired concentrations.

### 3.2. Raw 264.7 Cell Culture

Raw 264.7 cells (murine monocyte/macrophage cell line) were cultured in Dulbecco’s modified eagle medium (DMEM) supplemented with 10% foetal bovine serum (FBS) with 100 μM Ald for 20 h and then with 10 μg/mL LPS for an additional 24 h. Each isoprenoid was separately administered. LYC was given 2 h before the treatment with Ald, while GGOH and PHY were administered to cells simultaneously with Ald. At the end of the incubation period the supernatants were collected for NO and cytokines analyses, while cells were pelleted for programmed cell death, mitochondrial membrane potential and NALP3 expression assays.

### 3.3. Evaluation of Programmed Cell Death

The programmed cell death of Raw 264.7 cells was monitored by flow cytometry using double staining with Annexin V-FITC and Propidium Iodide (Apoptosis Detection Kit, Immunostep, Salamanca, Spain), according to the manufacturer’s instructions.

Fluorescence was acquired with Cyan ADP cytometer and Summit software (Beckman Coulter, Fort Collins, CO, USA) and then analysed with FlowJo software (version7.6, TreeStar Inc., Ashland, OR, USA). This technique was used to assess the effect of treatments on cell viability. Debris were excluded from the plot based on the scatter (FSC *vs.* SSC), and apoptotic (Annexin V positive, A+; Propidium Iodide negative PI− and positive PI+) and necrotic (Annexin V negative, A−; Propidium Iodide positive PI+) cells were characterized based on the fluorescence emitted.

### 3.4. Mean Fluorescence Intensity (MFI)

MFI was analysed with flow cytometry (Rhodamine 123, Sigma-Aldrich, MO, USA): results are expressed as mean fluorescent intensity (MFI) of Rhodamine 123 of three independent experiments. Fluorescence was acquired with CyAn ADP analyzer and Summit software (Beckman Coulter, Brea, CA, USA), then analysed with FlowJo software (version 7.6, Treestar Inc., Ashland, OR, USA).

### 3.5. Measurement of Cytokines in Cell Culture Supernatants

Cytokine levels were measured in Raw 264.7 cell culture supernatant samples by performing a bead-based multiplex immunoassay (23 mouse-Bio-Plex assay; BioRad Laboratories, Milan, Italy), following the manufacturer’s instructions. Data from the reactions were acquired using the Bio-Plex 200 reader, while a digital processor managed data output and Bio-Plex Manager^®^ 6.0 software presented data as Median Fluorescence Intensity and concentration (pg/mL) as well (BIO-RAD Laboratories, Milano, Italy).

### 3.6. Nucleotide-Binding Oligomerization-Domain Protein-Like Receptors 3(NALP3) RNA Isolation and Real Time-PCR (Polymerase Chain Reaction)

Total RNA was extracted from cells using the RNAqueous^®^-Micro Kit (Ambion^®^, Padova, Italy) and reverse transcribed into cDNA with a High Capacity cDNA Reverse Transcription Kit with RNase Inhibitor (Applied Biosystems^®^, Monza, Italy). Semi-quantitative real-time PCR was performed by using Taqman Gene Expression Assays for mouse *ACTB* (Mm01205647_g1) and *NALP3* (Mm00840904_m1) gene (Applied Biosystems^®^, Monza, Italy) in Applied Biosystems 7500 Fast real-time PCR System. The amplification efficiency of these primers was established beforehand and found to be comparable. The PCR amplification cycle was “standard mode”: after denaturation at 96 °C for 10 min, 40 PCR cycles were performed composed by 15 s at 95 °C and a final melting step for 1 min at 60 °C. All samples were analysed in triplicate.

### 3.7. Nitric Oxide Production on Raw 264.7 Cell Cultures

Nitric oxide production was assayed on supernatants using Griess Reagent (Sigma-Aldrich, Milan, Italy). The assays were performed in triplicate following the manufacturer’s indications.

### 3.8. Data Analysis

NO data are reported as percentage and compared to that of the Ald + LPS group, considered as 100% of NO production.

Other results are expressed as the mean ± standard deviation (SD). Statistical significance was calculated using a one-way analysis of variance (ANOVA) and Bonferroni post-test correction in the case of multiple comparisons. Analysis was performed using GraphPad Prism software (version 5.0) (GraphPad Software, Inc., La Jolla, CA, USA).

## 4. Conclusions

Lycopene, was not able to affect all the evaluated markers. Indeed, it exhibited only partial anti-oxidant and anti-inflammatory properties; although LYC treatment decreased pro-inflammatory cytokines release and NO production, it was not able to improve either cell viability or mitochondrial dysfunction. At present the only plausible hypothesis we can formulate is that the different activity of lycopene (C_40_H_56_), a tetraterpene assembled from eight isoprene units, could depend on its chemical features, which are different from those of Phytol (C_20_H_40_O), an oxygenated acyclic diterpene, and of the diterpene alcohol geranylgeraniol (C_20_H_34_O).

However, our findings demonstrated that phytol and geranylgeraniol were able to restore the deregulated mevalonate pathway and to antagonize the effects of the biochemical block, through their anti-inflammatory and anti-oxidant activities. Indeed, these isoprenoids, succeeded in counteracting the mitochondrial dysfunction and subsequent apoptosis, induced by the block of the pathway, by decreasing all the evaluated markers of both inflammation and oxidative stress.

We can therefore speculate that the anti-inflammatory activity shown by phytol and geranylgeraniol may be supported by their anti-oxidant properties.

These findings could, finally, be the starting point to better understand the activity of exogenous isoprenoid compounds and to consider them as potential novel therapeutic tools for the still orphan drug disease MKD.
